# Overriding TKI resistance of renal cell carcinoma by combination therapy with IL-6 receptor blockade

**DOI:** 10.18632/oncotarget.19420

**Published:** 2017-07-21

**Authors:** Kei Ishibashi, Tobias Haber, Ines Breuksch, Susanne Gebhard, Takashi Sugino, Hitoshi Kubo, Junya Hata, Tomoyuki Koguchi, Michihiro Yabe, Masao Kataoka, Soichiro Ogawa, Hiroyuki Hiraki, Tomohiko Yanagida, Nobuhiro Haga, Joachim W. Thüroff, Dirk Prawitt, Walburgis Brenner, Yoshiyuki Kojima

**Affiliations:** ^1^ Department of Urology, Fukushima Medical University, Fukushima, Japan; ^2^ Department of Urology, Johannes Gutenberg University Medical Center, Mainz, Germany; ^3^ Department of Gynecology and Obstetrics, Johannes Gutenberg University Medical Center, Mainz, Germany; ^4^ Department of Pathology, Shizuoka Cancer Center, Shizuoka, Japan; ^5^ Advanced Clinical Research Center, Fukushima Medical University, Fukushima, Japan; ^6^ Center for Pediatrics and Adolescent Medicine, Johannes Gutenberg University Medical Center, Mainz, Germany

**Keywords:** renal cell carcinoma, tyrosine kinase inhibitor, resistance, IL-6, tocilizumab

## Abstract

Metastatic renal cell carcinoma (RCC) is a tumor entity with poor prognosis due to limited therapy options. Tyrosine kinase inhibitors (TKI) represent the standard of care for RCCs, however a significant proportion of RCC patients develop resistance to this therapy. Interleukin-6 (IL-6) is considered to be associated with poor prognosis in RCCs. We therefore hypothesized that TKI resistance and IL-6 secretion are causally connected. We first analyzed IL-6 expression after TKI treatment in RCC cells and RCC tumor specimens. Cell proliferation and signal transduction activity were then quantified after co-treatment with tocilizumab, an IL-6R inhibitor, *in vitro* and *in vivo*. 786-O RCC cells secrete high IL-6 levels after low dose stimulation with the TKIs sorafenib, sunitinib and pazopanib, inducing activation of AKT-mTOR pathway, NFκB, HIF-2α and VEGF expression. Tocilizumab neutralizes the AKT-mTOR pathway activation and results in reduced proliferation. Using a mouse xenograft model we can show that a combination therapy with tocilizumab and low dosage of sorafenib suppresses 786-O tumor growth, reduces AKT-mTOR pathway and inhibits angiogenesis *in vivo* more efficient than sorafenib alone. Furthermore FDG-PET imaging detected early decrease of maximum standardized uptake values prior to extended central necrosis.

Our findings suggest that a combination therapy of IL-6R inhibitors and TKIs may represent a novel therapeutic approach for RCC treatment.

## INTRODUCTION

Renal cell carcinomas (RCC) account for about 85 percent of renal cancers and a quarter of the patients present with advanced disease, including locally invasive or metastatic renal cell carcinoma [1]. Currently, therapy targets the vascular endothelial growth factor (VEGF) and the mammalian target of rapamycin (mTOR) pathways represents the standard of care in metastatic RCC. Multitargeted tyrosine kinase inhibitors (TKIs) lead to clearly prolonged overall and progression-free survival [2]. The TKI sorafenib inhibits VEGFR-2, VEGFR-3, the platelet-derived growth factor receptor family (PDGFR-β and Kit) as well as both C-RAF and B-RAF [3]. Sunitinib is a highly potent, selective inhibitor of VEGF-R types 1 to 3, PDGF-Rα, and PDGFR-β [4]. Pazopanib also inhibits all the VEGFR subtypes and the PDGFR subtypes. In addition, it inhibits the fibroblast growth factor receptor, as well as transmembrane glycoprotein receptor tyrosine kinases [5]. However, despite the development of many types of TKIs, their effects are still limited and have been shown to be not curative [6]. A number of approved molecular targeted agents allow the sequential use of these drugs as empirical standard of care, although the optimum order of application has not been defined [7, 8]. Moreover, treatment has been associated with the development of resistance after a median of 6–15 months [9]. Alternative signaling pathway activation has been shown to be responsible for this resistance for most of the listed targeted RCC therapies. According to these studies, one of the central effects involved in TKI resistance [10] is probably due to sphingosine kinase-1 (SK1) activation, that stabilizes HIF-1α via enhanced AKT and ERK signaling [11]. To date it is not clear which mechanisms lead to these enhanced signaling activities. The finding that IL-6 seems to be involved in the development of TKI resistance [12, 13] suggests that cytokines are important in this process. IL-6-induced AKT phosphorylation activates mTOR that consequently activates its downstream targets p70S6 kinase (p70S6K), 40S ribosomal protein S6 (S6RP) and the eukaryotic initiation factor 4E binding protein-1 (4EBP1), that control mRNA translation and protein synthesis [14]. Consequently, IL-6-induced activation of AKT is involved in protection against apoptosis, as well as in enhanced proliferation in some cancer cells [15–17].

Tocilizumab, a humanized antihuman IL-6 receptor (IL-6R) antibody, is currently available as one of the therapeutically effective reagents against inflammatory diseases such as rheumatoid arthritis [18]. We have previously reported the effect of a combination therapy using the antihuman IL-6R antibody together with IFN-α, that suggests a novel therapeutic approach for the treatment of RCC [19].

In the presented study, we analyzed the impact of cytokines during TKI treatment of RCC cells. We show that the autocrine secretion of IL-6 induced by TKIs-stimulation causes the activation of AKT, mTOR and STAT3, which consequently lead to VEGF expression in 786-O RCC cells. We also show that a combination therapy with tocilizumab, a humanized anti-IL-6 receptor antibody, and TKI can effectively suppress 786-O RCC tumor growth.

## RESULTS

### TKIs induce IL-6 secretion by 768-O cells

**Figure 1:**
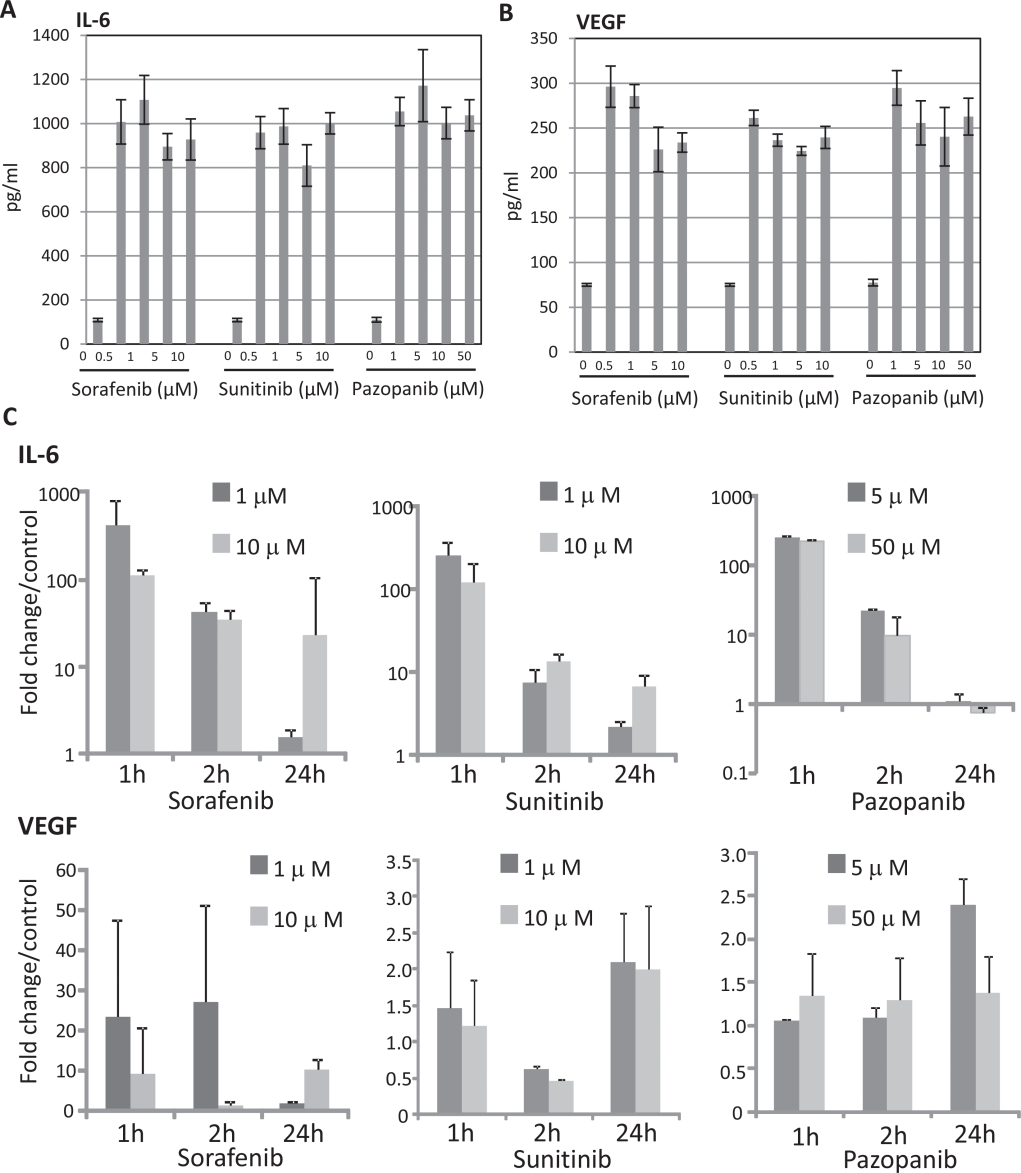
TKI-induced IL-6 and VEGF expression in RCC cells RCC cell line 786-O was cultured with sorafenib, sunitinib or pazopanib for one hour, and analyzed by VersaMAP Development System concerning the secretion of cytokines. Demonstrated are cytokines with clear changes in secretion compared to untreated cells: IL-6 (**A**), and VEGF (**B**). Untreated cells served as control. Relative mRNA expressions (**C**) of TKI treated (two concentrations, three time points) compared with those of non-treated cells are indicated. All TKIs up-regulate *IL-6* and *VEGF* mRNA expression up to 400 fold (for *IL-6*) and 25 fold (for *VEGF*), irrespective of their concentration.

Comprising all cytokines analyzed, IL-6 and VEGF secretion by 786-O cells were remarkably enhanced after TKI treatment. Sorafenib, sunitinib and pazopanib induced a strong (10-fold) enhanced secretion of IL-6 in 786-O cells, even at a low concentration (Figure 1A). VEGF secretion was also strongly increased in 786-O cells after stimulation with each of the three TKIs (Figure 1B). In all other RCC cell lines tested, TKI treatment caused no significant changes in cytokine secretion (data not shown).

### Gene expression of IL-6 and VEGF in 786-O cells

To find out if the enhanced secretion of IL-6 and VEGF proteins in 786-O cells corresponds to an enhanced mRNA level of the respective genes, we investigated the influence of TKIs on *IL-6* and *VEGF* expression. All TKIs used (sorafenib, sunitinib and pazopanib), induced increased *IL-6* and *VEGF* mRNA expression one hour after stimulation, with exception of pazopanib treatment, that did not result in a significant *VEGF* induction. Increased mRNA expression levels after TKI treatment sustained until 24 hours after TKI stimulation (Figure 1C) with the exception of *IL-6* expression after pazopanib treatment.

### IL-6 expression in RCC surgical specimens

**Figure 2:**
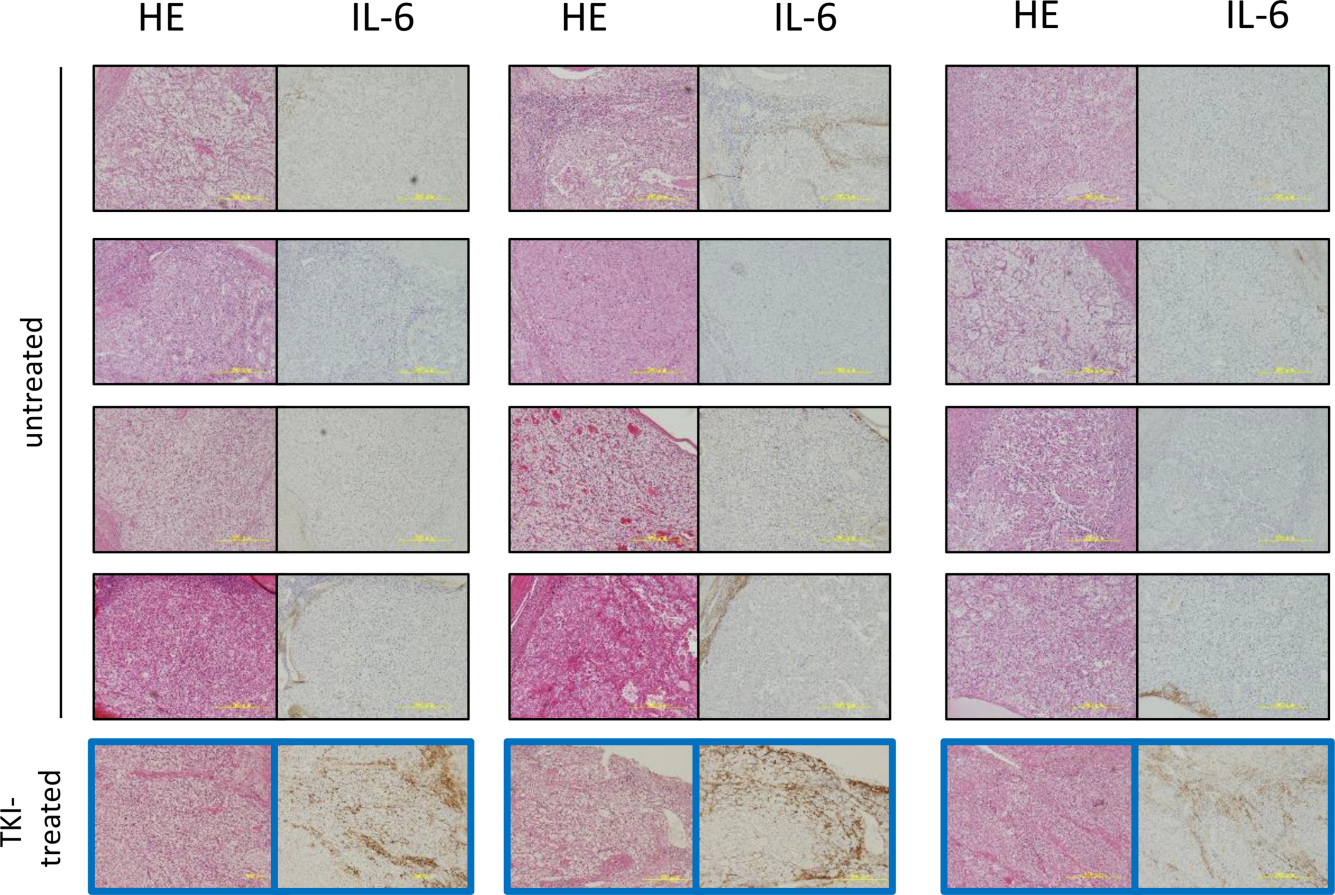
Immunohistochemical staining of IL-6 in specimens of RCC patients treated with TKIs or without therapy before nephrectomy HE and immunohistochemical staining of IL-6 of surgical RCC specimens diagnosed with over pT3 clear cell renal cell carcinoma. Three patients were treated with TKIs for two months prior to surgery. The surgical specimens from the three patients showed strong IL-6 expression (Figure 2 framed blue). In contrast, all of 12 patients without TKIs treatment before surgery did not show IL-6 expression in the tumor parenchyma.

We retrospectively analyzed the expression of IL-6 in RCC specimens from 15 patients who underwent radical nephrectomy. Among the 15 patients, 3 were neoadjuvantly treated with TKIs (two with sorafenib, one with sunitinib) before they were referred to our institution for surgery. The surgical specimens from these 3 patients showed strong IL-6 expression. In contrast, weak or no IL-6 expression was observed in specimens from non-TKIs treated patients (Figure 2).

### Impact of TKI stimulation on the IL-6 signaling

**Figure 3:**
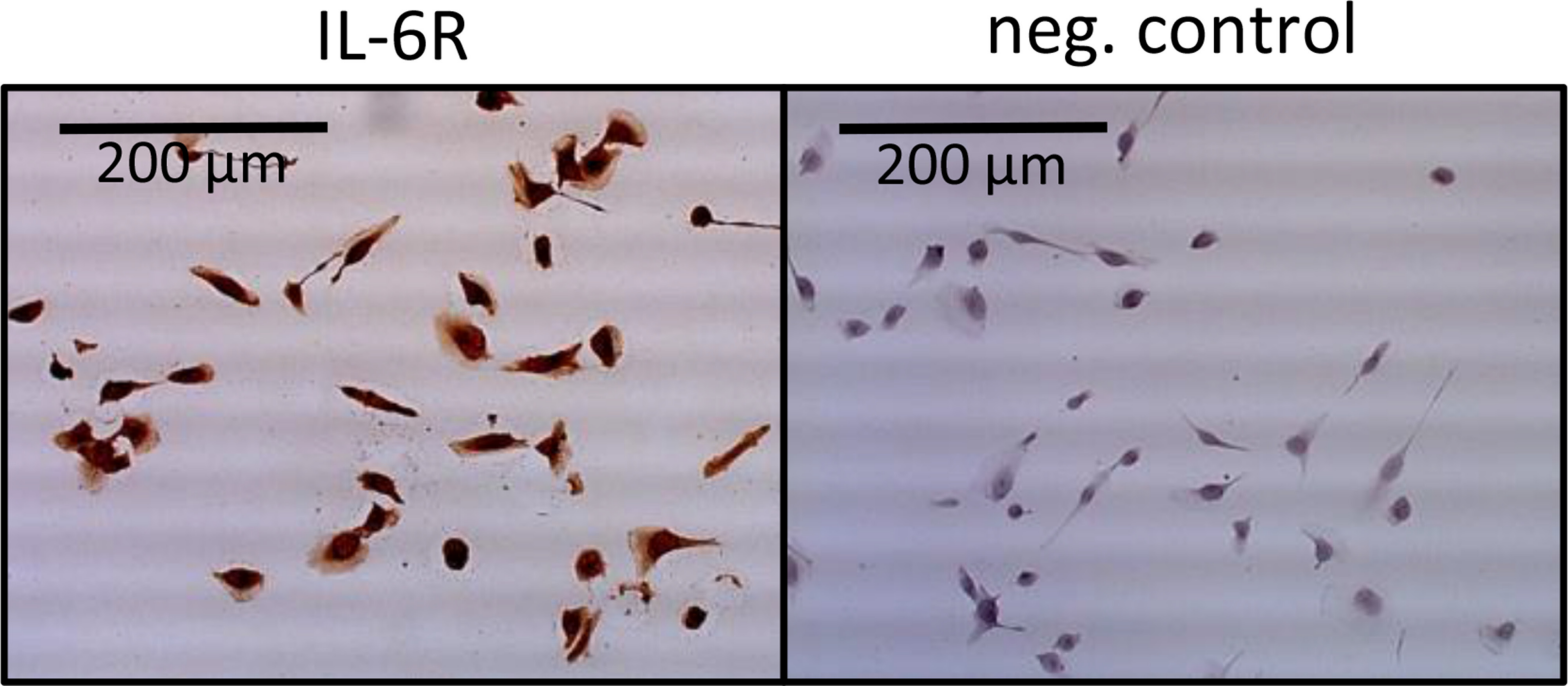
Immunohistochemical staining of IL-6R in 768-O cells Cells show a clear positive IL-6R staining, whereas the negative control remains unstained.

**Figure 4:**
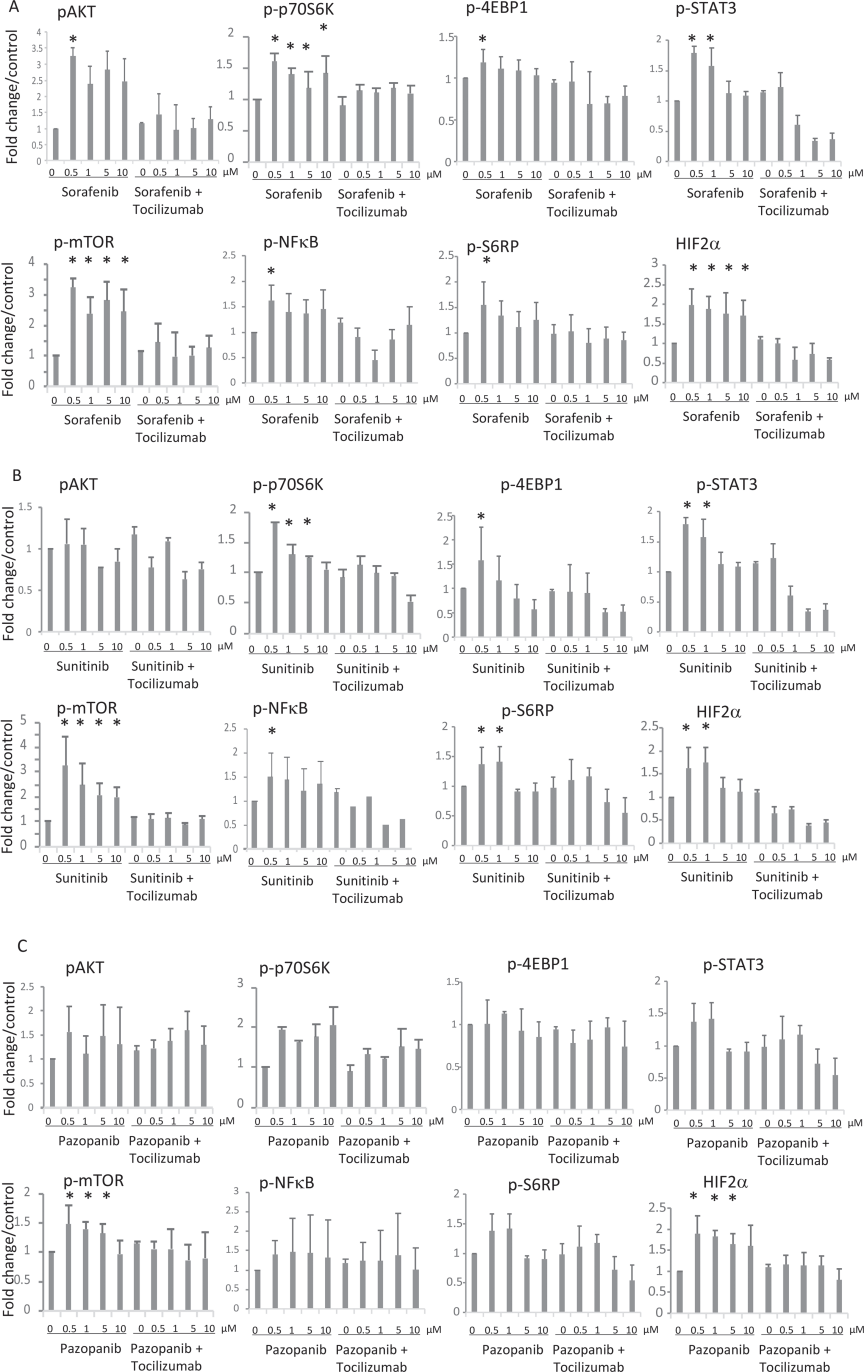
Effects of IL-6 signaling blockade on activity of the AKT-mTOR pathway after (**A**) sorafenib, (**B**) sunitinib or (**C**) pazopanib treatment. Influence of tocilizumab (50 μg/ml) on activation of AKT-mTOR pathway in 786-O cells after treatment with TKI was determined by Western blot. The concentrations of TKIs are indicated on the X axis of each graph. In combinational treatment (right part of each graph) tocilizumab was used in a concentration of 50 μg/ml. The column labeled by 0 μg/ml in the right part of each graph represents treatment with tocilizumab alone. Phosphorylation of AKT, mTOR, 4EBP1, S6RP, NFκB, and STAT3 was enhanced after TKI treatment at concentration of 0.5 μM. HIF-2α was enhanced by treatment with TKIs in all concentrations used. Tocilizumab neutralized these effects. The results are presented as the relative mean value ± standard deviation of three independent analyses, each in triplicate, related to untreated control cells. *p < 0.05 compared with the control cells.

Since we observe a high expression of IL-6R in 786-O cells (Figure 3), we studied the impact of TKI stimulation on the associated IL-6 signaling pathway. We examined the pathway activation by Western blot, monitoring phospho-AKT, phospho-mTOR, phospho-4EBP1, phospho-S6RP, phospho-p70S6, phospho-NFκB, phospho-STAT3 and HIF-2α in 786-O cells treated with TKIs in combination with or without the blocking antihuman IL-6R antibody tocilizumab. In 786-O cells we found a concentration depend enhanced phosphorylation of AKT, mTOR, 4EBP1, S6RP, p70S6, NFκB and STAT3 after treatment with all of the TKIs tested, with exception of 4EBP1 and S6RP after pazopanib treatment. Sorafenib in a concentration of 0.5 μM significantly activated all investigated signaling molecules. STAT3 was additionally activated by sorafenib in a concentration of 1 μM, whereas the activity of p70S6K, mTOR and HIF2α was enhanced after sorafenib treatment in all concentrations investigated. Sunitinib treatment in a concentration of 0.5 μM resulted in an enhanced, but slightly non-significant, activity of AKT and a significant enhanced activity of all other signaling molecules investigated. Additionally, sunitinib induced activation of p70S6, STAT3, mTOR, S6RP and HIF-2α in a concentration of 1 μM. In a concentration of 5 μM p70S6 and in a concentration of 10 μM mTOR was activated. Although all signaling mediators with exception of S6RP were activated by pazopanib treatment, the enhancement was only significant in case of AKT by treatment with 0.5 μM and of mTOR and HIF-2α in concentrations between 0.5 and 5 μM. The enhanced phosphorylation of the named proteins after TKI treatment at all concentrations used was also associated with enhanced HIF-2α protein amounts, one of the transcription factors of VEGF (Figure 4). Treatment with the IL6-R blocking tocilizumab abolished the effect of the TKIs on the activation of AKT, mTOR, 4EBP1, S6RP, p70S6, NFκB, STAT3 and HIF-2α (Figure 4), arguing that the observed TKI effects depend on the enhanced IL-6 signaling in 786-O cells. In addition, treatment with tocilizumab alone did not influence the activation of any of the signaling molecules investigated (Figure 4).

### Alteration on susceptibility to TKIs by IL-6 pathway blockade

**Figure 5:**
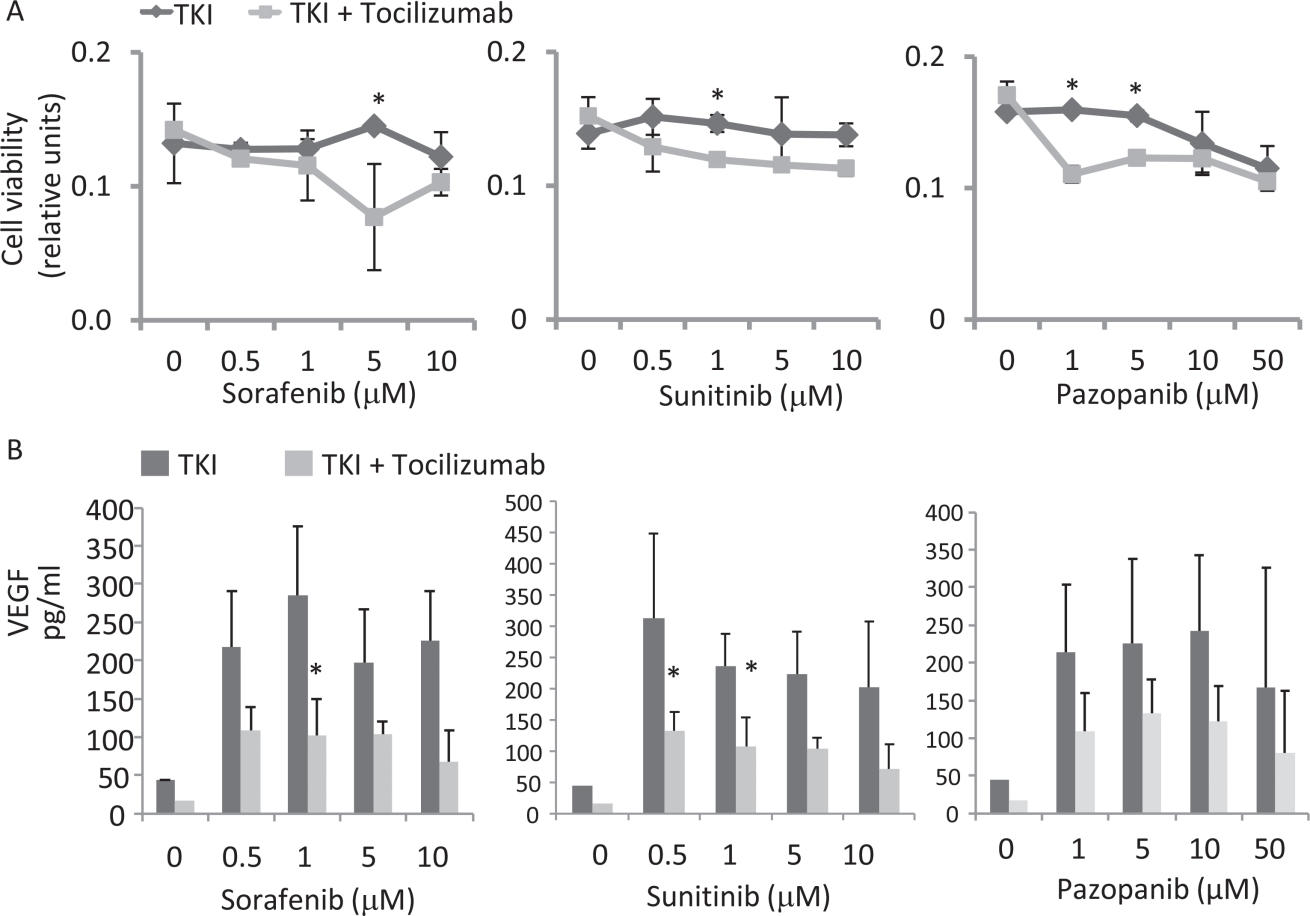
Cell viability as well as VEGF secretion after IL-6 signaling inhibition (**A**) Antihuman IL-6R antibody, tocilizumab (50 μg/ml), was used for the inhibition of IL-6 signaling. Cell count was determined by an MTT assay. Tocilizumab significantly increased susceptibility to TKIs, especially at low dosage of 5 μM for sorafenib, 1 μM for sunitinib and 1 and 5 μM for pazopanib. *p < 0.05 for two-tailed paired *t*-test compared with combination with tocilizumab and TKIs. (**B**) Tocilizumab inhibits low dosage TKI-induced VEGF expression, determined by ELISA. *p < 0.05 for two-tailed paired *t*-test compared with the TKI treated cells.

Our data suggested that 786-O cells are resistant to a sole TKI treatment and most likely proliferate due to the increased IL-6 secretion. To study the impact of IL-6 on cell proliferation, we determined the effect of TKIs on 786-O cells under the blockade of IL-6 signaling by tocilizumab in a MTT assay. TKIs in low doses and tocilizumab alone (value of 0.93±0.09, relative to control) did not influence the cell count. The addition of tocilizumab to TKIs results in a significant reduction of the 786-O cell count (Figure 5A).

We quantified the protein secretion levels of VEGF by ELISA in 786-O cells treated with tocilizumab. As suggested by the STAT3 and HIF-2α Western blot analyses described above, sorafenib treatment resulted in a strongly enhanced VEGF secretion. Treatment with the other TKIs sunitinib or pazopanib also induced an enhanced VEGF secretion in this cell line (Figure 5B). However blocking of the IL-6R by tocilizumab attenuated the effect of sorafenib and sunitinib significantly (*p* = 0.035 for sorafenib 1 μM, *p* = 0.019 for sunitinib 1 μM and *p* = 0.044 for sunitinib 5 μM).

### *In vivo* growth inhibitory effect of combination therapy with tocilizumab and low-dose sorafenib

**Figure 6:**
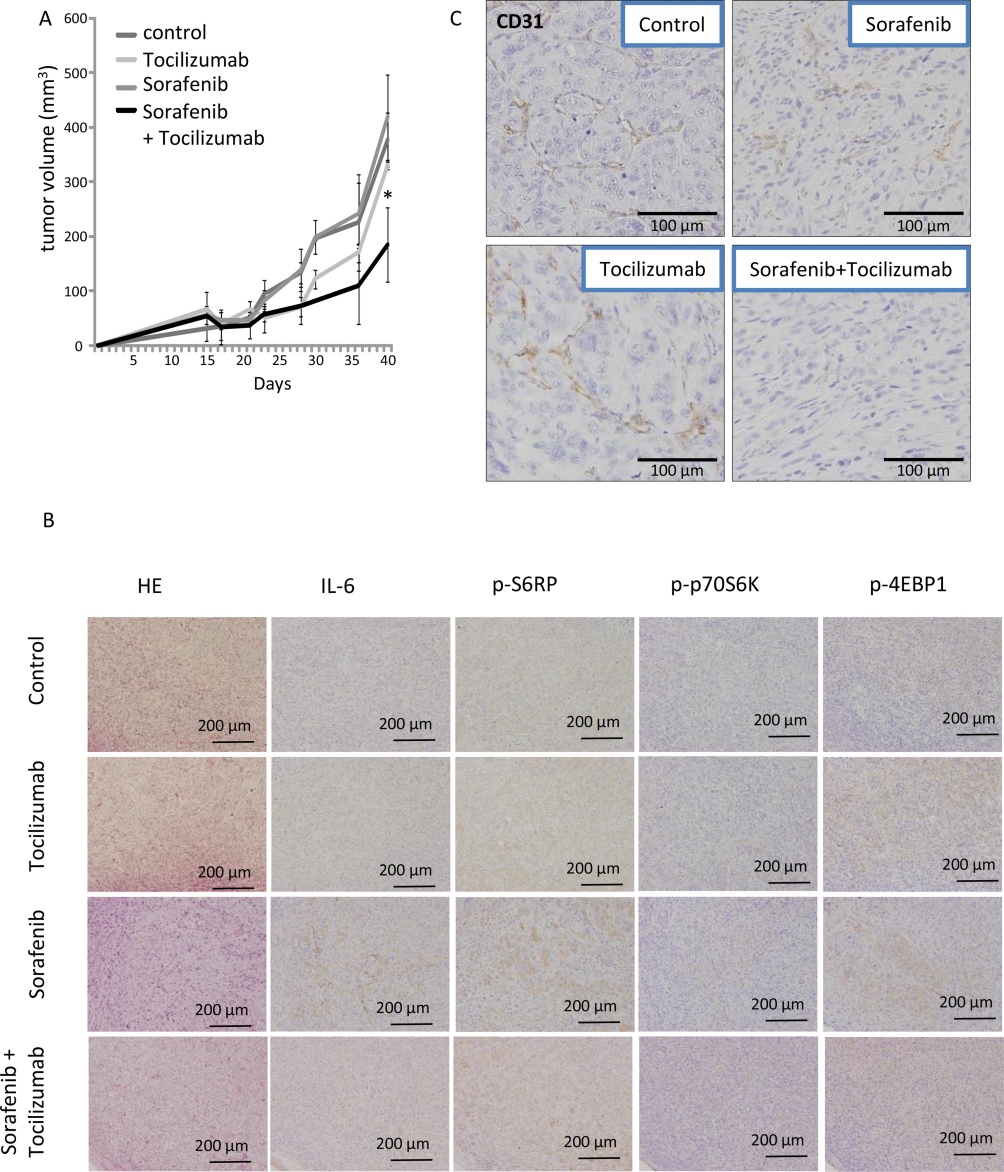
Effect of combination therapy with sorafenib and tocilizumab on a nude mice xenografts model of 786-O cells Mice were treated three times a week with tocilizumab (100 μg i.p.) or control PBS (*n* = 5 each) followed by daily sorafenib administration at 10 mg/kg/day as described elsewhere [54]. Phosphate-buffered saline (PBS) was used in the control (non-treated) mouse group (*n* = 5). Tocilizumab alone was used in another group of mice (*n* = 5). Mice were sacrificed after 40 days and tumors were analyzed. (**A**) Time course of tumor volume was analyzed by ellipsoid volume formulas (pi/6 x L x W x H). The tumor volumes by low-dose sorafenib + tocilizumab therapy were significantly decreased compared to sorafenib therapy alone (*p* < 0.05). A repeated experiment yielded similar results. (**B**) Endothelial cells of xenografts were visualized by an immunohistochemical staining of CD31. Sorafenib or tocilizumab administration alone partially inhibited angiogenesis in the tumor as demonstrated by reduced CD31 levels. The combination therapy with sorafenib and tocilizumab strongly inhibited the angiogenesis. (**C**) Signaling pathways of xenografts were analyzed immunohistochemically. Sorafenib at a low dose of 10 mg/kg/day induced activation of S6RP, p70S6K and 4EBP1 of mTOR pathway. Tocilizumab administration resulted in no remarkable morphological changes. The combination therapy diminished the effect induced by sorafenib alone on the activation of mTOR pathway.

To confirm that tocilizumab improves the effect of low dose TKIs in the MTT assay, we employed a nude mice xenograft model, applying low dose sorafenib with or without tocilizumab. The growth of tumors in athymic mice receiving a combination therapy with tocilizumab and sorafenib was retarded in comparison to those in the sorafenib-alone and non-treated (PBS) groups (Figure 6A). In the earlier stage of the challenge (up to 30 days), growth of tumors in the group treated with tocilizumab alone seemed to be retarded, yet there was no significant difference to the control / sorafenib treated groups at the time of sacrifice (41 days). However, the tumor volume in the group with combination therapy (sorafenib and tocilizumab) was significantly lower compared with other groups at the end of the study (*p* = 0.044).

**Figure 7:**
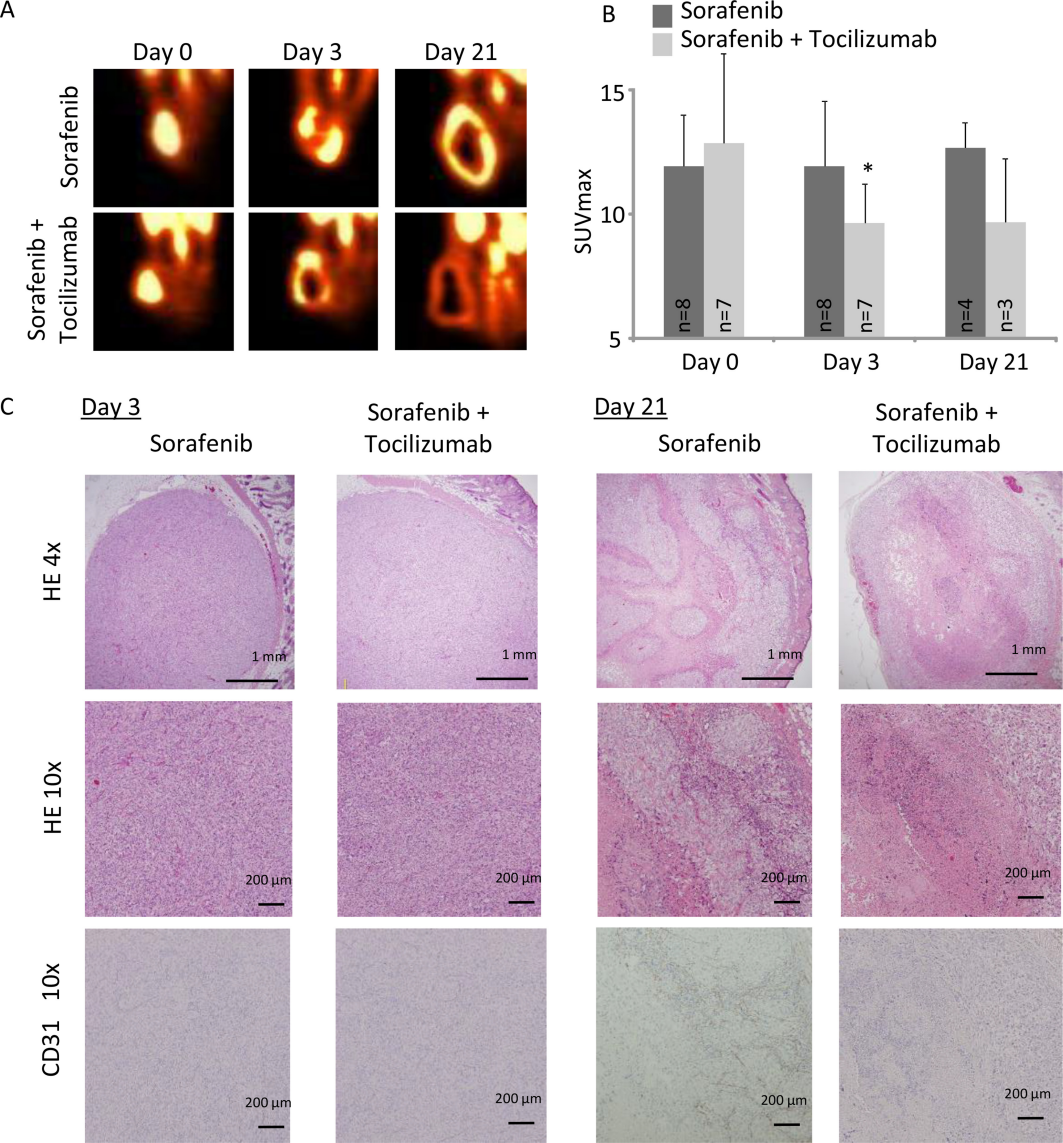
A nude mice xenograft model of 786-O cells was evaluated by FDG-PET imaging during a time course of 3 and 21 days Mice were treated with sorafenib alone (30 mg/kg/day) or in combination with tocilizumab (100 mg three times a week). (**A**) Sorafenib as well as combination therapy with sorafenib and tocilizumab lead to a decrease of the viable region in the central area of the tumor. (**B**) SUVmax was significantly decreased in the tumor in the combination therapy compared with sorafenib alone at day 3 (SUVmax 9.6 vs. 11.9, *p* = 0.04). At day 21, FDG-PET imaging showed that the SUV value remained low in the tumor with the combination therapy, although value difference was not significant compared with sorafenib treatment alone (*p* = 0.10). (**C**) RCC in nude mice were analyzed by HE and endothelial cell staining (CD31) on day 3 and day 21. Tumors from mice treated with sorafenib, as well as with the combination therapy, showed no remarkable change on day 3 of challenge. CD31 positive cells were only marginal in both groups. On day 21 tumors from mice treated with sorafenib, as well as with combination therapy, showed central necrosis in the tumor. However, tumors from mice treated with sorafenib alone retain a viable tumor area at the central tumor region and show CD31 positive cells among the viable tumor cells.

Subsequently, tumors were processed for microscopic morphological description. The tumors from the control mice displayed a clear cell-type, specific for renal cell carcinoma. Immunohistochemical examination confirmed the absence of IL-6 and negligible activation of mTOR signaling in the tumor cells of non-treated mice. No remarkable differences were observed in the tumors treated with tocilizumab alone. Tumor sections obtained from the mice after sorafenib-only treatment showed enhanced expression of p-S6RP, p-p70S6K and p-4EBP1 as a result of an activation of the mTOR pathway. In contrast, the combination therapy with sorafenib and tocilizumab blocked the enhanced activation of the mTOR signaling cascade and showed lower amounts of p-S6RP, p-p70S6K and p-4EBP1 compared to tumors treated with sorafenib-only (Figure 7B). The vascularization of the xenograft tumors was also dependent on the treatment. Tumors of mice treated with sorafenib or tocilizumab alone showed a slightly reduced vascularization of the tumor, determined by CD31 staining (Figure 6C). However, a remarkable reduction of angiogenesis (compared with PBS-control, sorafenib- or tocilizumab-only) was observed when mice were treated with tocilizumab in combination with low dose sorafenib (Figure 6C).

### FDG-PET imaging

**Figure 8:**
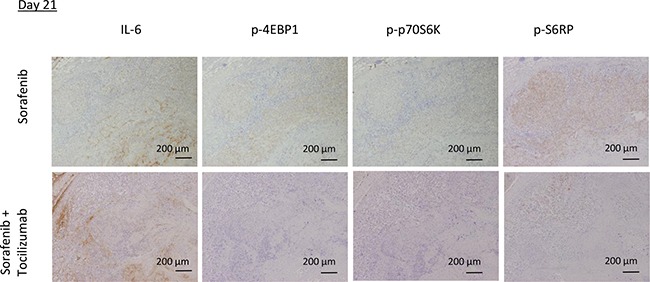
mTOR pathway activity in mouse tumor specimens after sorafenib treatment in combination with tocilizumab The mTOR pathway in the tumors was immunohistochemically analyzed on day 21. Tumors from mice treated with sorafenib showed phosphorylation of mTOR pathway components 4EBP1, p70S6K and S6RP, correlating with IL-6 expression. In contrast, tumors from mice of the combination therapy group showed suppressed activation of the mTOR pathway.

To investigate TKI resistance in RCC and the possibility of its prevention by combination therapy with sorafenib and tocilizumab *in vivo*, we employed FDG-PET imaging. Tumor viability, determined by maximum standardized uptake value (SUVmax), of mice treated with sorafenib alone remained unchanged with a tendency to enhanced viability at day 21 (Figure 7A). In the mice treated by combination therapy, tumor viability was significantly decreased after 3 days compared to the mice treated with sorafenib alone (*p* = 0.04). Also on day 21, the tumor viability of mice treated with sorafenib in combination with tocilizumab was clearly lower than that of sorafenib treated mice, although this difference was not significant (Figure 7B). No histopathological differences were found on day 3 between the two groups, despite decreased CD31 positive cells and significantly lower signal intensity on the FDG-PET in the mice treated with combination therapy (Figure 7C). After 21 days, hematoxylin-eosin staining showed extensive necrosis, both in mice treated with sorafenib monotherapy and sorafenib / tocilizumab combination therapy (Figure 7C). However, there still remain viable tumor cells within the necrotic area of tumors in the mice treated with sorafenib monotherapy. Also, the CD31 positive cells increased again in the tumor region treated with sorafenib along on the day 21. In contrast, when tocilizumab was given in combination with sorafenib, the tumors showed extensive central necrosis with absence of CD31 positive cells (Figure 7C). Immunohistochemical examination confirmed suppressed activation of S6RP, p70S6K and 4EBP1 in the tumors after combination therapy, although IL-6 was detected (Figure 8).

## DISCUSSION

The present study demonstrates that low concentration TKI treatment induces IL-6 and VEGF expression and secretion only in 786-O cells, whereas other RCC cell lines were not affected by TKI concerning cytokine secretion. The finding that in all cases of neoadjuvant TKI treated patient's RCC specimens IL-6 was highly expressed, whereas in patients without TKI treatment no or only very low IL-6 expression was detectable, suggests that the cell line 786-O reflects the situation *in vivo* and we therefore used it for all further investigations. 786-O cells differ from the other cell lines by simultaneous mutations of VHL and HIF-1α [20]. HIF-1α and HIF-2α are known to reciprocally influence each other concerning VEGF expression and in renal cancer, VEGF secretion particularly is regulated by HIF-2α [21]. Due to the VHL mutation in 786-O cells, HIF-2α is persistently highly expressed [22] and due to the loss of HIF-1α, the reciprocal interaction between HIF-1α and HIF-2α probably is abolished, suggestively leading to a higher secretion of VEGF. This coincidence may cause the here described strong IL-6-VEGF vicious circle especially in 786-O cells.

IL-6 is a pleiotropic cytokine with widespread effects on hematopoietic lineages [23, 24] and is considered to be a key mediator of inflammation [25]. Dysregulation of IL-6 signaling contributes to the onset and maintenance of several diseases including some types of cancer, *i.e*. multiple myeloma, gastric cancer and prostate cancer [26, 27]. IL-6 induces activation of the PI3K/AKT pathway, and is involved in protection against apoptosis and in enhanced proliferation of multiple myeloma cells [15, 16]. In this context it is not surprising that inflammation dependent secretion of IL-6 induces TKI resistance in lung cancer [28]. It has been described, that in RCC circulating cytokine levels, including IL-6, are increased in advanced RCC patients treated with sunitinib from baseline values before tumor progression [29]. Interestingly, all three clinical specimens we obtained from RCC patients who were treated with TKIs prior to surgery showed strong IL-6 expression in the tumor parenchyma, although the number of the patients was quite small. One can assume that tumor necrosis due to TKI treatment causes inflammatory changes and leads to cytokine secretion. Inflammatory cytokines such as IL-6 might then affect the viability of the cells which survive after TKI treatment.

**Figure 9:**
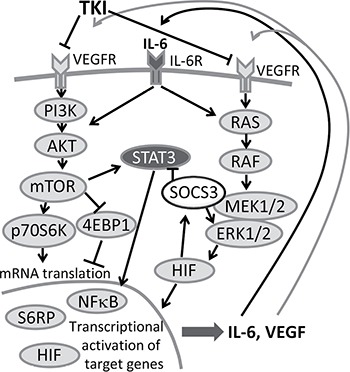
Feedback loop in TKI therapy IL-6 and VEGF activate AKT-mTOR and the STAT3 signaling cascade, consequently leading to enhanced secretion of IL-6 and VEGF. Secreted IL-6 and VEGF can again re-activate tumor cells and thus trigger tumor progression. A combination therapy of TKI together with an anti-IL-6R antibody could inhibit this amplifier circle.

We found that the elevated IL-6 secretion is correlated with an increased VEGF secretion, suggesting a functional coupling of the two events. A mandatory requirement for such a loop is the expression of the IL-6 receptor, which we observed in 786-O cells in our study. It is known, that in response to IL-6R stimulation, cytoplasmic STAT3, an independent prognostic marker in RCC [30], gets activated. STAT3 is known to play an important role in tumor progression of several high-malignant cancer entities. In intrahepatic cholangiocarcinoma STAT3 overexpression negatively correlates with the outcome of the patients [31], and in breast and lung cancer STAT3 has a key role in metastatic processes [32, 33]. Phosphorylation of STAT3 triggers a transcriptional response favoring survival, proliferation and angiogenesis. Phosphorylated STAT3 rapidly accumulates in the nucleus [34] and leads to the production of VEGF [35, 36]. The activated STAT3 can increase NFκB activity and NFκB itself is an important mediator for activation of the IL-6 gene [37]. This activation cascade is effective within short time, since inhibition of NFκB results in a reduced IL-6 secretion after a period of one hour [38], a time period also selected in our study. Within the cell IL-6 is stored in vesicles and secreted after stimulation [39]. Apparently, IL-6 transcription seems to be simultaneously enhanced or degradation diminished. NFκB-triggered positive feedback for IL-6 signaling, also known as inflammation amplifier, induces various molecules to create a microenvironment which promotes cancer development [40]. In our study we show, that TKI treatment enhances IL-6 secretion, associated with activation of the AKT pathway including AKT, mTOR and p70S6K as well as STAT3 and NFκB activation and consequently VEGF secretion. This suggests a similar amplifier loop in renal cancer, whereby IL-6 is induced by TKIs in RCC cells, consequently up-regulating VEGF expression via AKT and STAT3 (Figure 9). The observation that the AKT-mTOR pathway is activated only when low concentrations of TKIs are used, although enhanced secretion of IL-6 and VEGF also occur when higher TKI concentrations are added, may be caused by the circumstance that not the AKT-mTOR pathway alone is responsible for the enhanced cytokine secretion, but also other mechanisms not detected in this study. This is in good accordance with the finding, that a higher VEGF baseline level is associated with less progression-free survival in sunitinib treated RCC patients [41, 42].

It has been reported, that IL-6-induced activation of the PI3K/AKT pathway is involved in protection against apoptosis, as well as in enhanced proliferation in some cancer cells [15–17]. Phosphorylated AKT activates downstream molecules like mTOR, a key factor in this pathway. STAT3 is directly phosphorylated by activated mTOR. STAT3, 4EBP1 and S6K1 act in concert to drive the angiogenic response through mTOR complex 1 (mTORC1) [43]. The mTORC1 activation causes protein synthesis, which induces cell proliferation, survival, motility and invasion leading to cancer initiation and progression [44]. On the other hand, mTOR complex 2 (mTORC2) again activates AKT and can induce the expression of HIF-2α [45], an anti-apoptotic transcription factor, highly important in tumorigenesis [46]. HIF-2α has been demonstrated to be more important in RCC tumorigenesis than HIF-1α, since blocking HIF-2α results in a strong reduction of RCC tumor formation and angiogenesis [47]. In our study activation of mTOR after low-concentration TKI treatment correlates clearly with an enhanced HIF-2α expression, probably inducing resistance to TKI treatment. However, the enhanced VEGF secretion observed after treatment with TKI in low concentration seems not only to be caused by HIF-2α, since HIF-2α was highly expressed after treatment in all TKI concentrations tested. In contrast, STAT3 was primarily enhanced after treatment with low TKI concentrations, corresponding to the high VEGF secretion, which is in good accordance with the fact that STAT3 is known to be able to induce VEGF secretion [48].

TKI treatment alone had no significant effect on proliferation of 786-O cells *in vitro*, probably due to the low concentrations used. Tocilizumab, an inhibitory IL-6R antibody, that is approved for rheumatoid arthritis, juvenile idiopathic arthritis, Castleman's disease, and Crohn's disease [49–52], inhibits IL-6 signaling. An additive treatment of RCC cells with TKI together with tocilizumab in the present study resulted in a reduced tumor cell proliferation. Additionally, VEGF secretion was reduced after combination treatment, compared to treatment with TKI alone. These results were significant only when the TKIs sorafenib and sunitinib were used, whereas in case of pazopanib we only could observe a tendency in the according direction. These combination effects were confirmed by our *in vivo* study using sorafenib together with tocilizumab, where the combination therapy enhanced anti-tumor effects of sorafenib in 786-O RCC cells in nude mice.

We have previously reported that IFN-α induces IL-6 in human RCC cell lines and that IL-6 plays a crucial role for resistance also against IFN-α treatment through suppressed STAT1 phosphorylation and induced phosphorylation of STAT3 and ERK [19]. Tocilizumab restored the inhibitory effect of IFN-α, *in vitro* and *in vivo*, in an IFN-α-resistant RCC cell line in which IL-6 was highly induced by IFN-α [19]. This further supports the possibility to block multiple treatment resistance mechanisms, induced by an enhanced IL-6 secretion, by inhibiting IL-6R, as we show here in RCC.

The 786-O tumors of mice treated with low doses of sorafenib monotherapy activated p70S6K, S6RP and 4EBP1, apparently leading to tumor cell proliferation under the insufficient effect of sorafenib. The combination with IL-6R antibody decreased this activation and significantly reduced the tumor volume compared to the sorafenib monotherapy.

In addition, as shown in the *in vivo* study using arterial spin-labeling magnetic resonance [53], the vascularity in the tumor was reduced only temporarily by sorafenib and returned to control levels on day 14–21 after treatment. The combination with IL-6R antibody prolonged the time of reduced vascularity, and we observed decreased vessels at the periphery of the lesion even on day 21 after combinational therapy with sorafenib and tocilizumab. This supports the idea, that the tumor angiogenesis is inhibited by an IL-6R blockade. We have previously reported that the inhibition of IL-6 signaling alone is insufficient to inhibit 786-O tumor growth *in vivo* [19]. Only the combination with sorafenib and tocilizumab inhibited tumor growth and angiogenesis. TKI resistance of renal cancer includes insensitivity against anti-proliferative effects of TKIs as well as resistance to anti-angiogenic TKI effects. The observed effect occurs after TKI treatment in low concentration. This can appear during dosage reductions in patients or after discontinuation of the TKI therapy, which may finally lead to TKI-resistance in anti-proliferative and anti-angiogenesis effects of further TKI treatment. In our cellular model, a combination of TKI with tocilizumab effectively circumvents TKI resistance. *In vivo* we confirmed this observation concerning angiogenesis. IL-6 secretion can activate VEGF expression and thus contribute to angiogenesis, cell proliferation and survival of tumor cells. We suggest that the IL-6 secretion of RCC cells might lead to renal cancer resistance towards TKI therapy, due to the discussed feedback loops. In this scenario, the combination of TKI with tocilizumab would have at least additive effects, since we observed early decline of SUVmax as well as earlier appearance and increase of the necrotic areas by FDG-PET after combination therapy.

In conclusion, the presented study demonstrates that sunitinib and sorafenib treatment in low concentrations leads to activation of AKT- and STAT3-signaling, consequently resulting in a secretion of IL-6 and VEGF. The latter then seems to result in an amplifier circle, which consecutive reduces sensitivity to TKIs. Inhibition of the IL-6 signaling by tocilizumab may block the amplifier circle and re-activate the anti-tumor activity of TKIs, both *in vitro* and *in vivo*. Our findings suggest that a combination therapy using an antihuman IL-6R antibody with TKIs may represent a novel therapeutic approach for the antiangiogenic treatment of RCC, also in the light of previously reported IFN-α effects.

## MATERIALS AND METHODS

### Cell lines/TKIs/tocilizumab

The human RCC cell lines 786-O, A489, Caki1 and Caki2, obtained from the American Tissue Culture Collection (ATCC), were cultured at 37°C in 5% CO_2_ using RPMI 1640 with 10% fetal bovine serum. Three kinds of TKIs, sorafenib, sunitinib and pazopanib, were purchased from LC Laboratories (Woburn, MA, USA), sorafenib additionally kindly provided from BAYER. The optimum concentration of tocilizumab was determined to be 50 mg/ml, as described previously [19].

### Patient surgical specimens

Surgical specimens were obtained from 15 patients with renal cell carcinoma who underwent radical nephrectomy for cT3N0M0 disease at the department of Urology, Fukushima Medical University, between 2010 and 2012. Mean patient age was 66 years. Patients who were diagnosed with clear cell renal cell carcinoma were included in this study. The research protocol was approved by the Ethics review committee of Fukushima Medical University (approval #2663). Among the 15 patients, two were neoadjuvantly treated with sorafenib, one with sunitinib for two months before surgery to reduce the tumor volume.

### Measurement of cytokines using VersaMAP development System

786-O cells were treated by TKIs at a final concentration of 0.5, 1.0, 5.0, 10.0 μM for sorafenib and sunitinib and 1.0, 5.0, 10.0, 50.0 μM for pazopanib. The supernatant was collected 1, 2, and 24 hours after TKIs stimulation. The amount of cytokine production (IL-6, VEGF, IL-1ra, IL-17, IL-19, IL-23, IL-18BPa, Leptin, HGF, Cript-1, HB-EGF, EGF) was measured by VersaMAP Development System (R&D systems, Minneapolis, MN, USA) according to the manufacturer's instructions. Briefly, 50 ml of Microparticle Cocktail was added to each well of the filter-bottomed microplate and 50 μl cell culture supernatant was added to each well. After a 2-hour incubation at room temperature on a microplate shaker, the liquid was removed and the plate was washed with wash buffer. Then 50 ml of Biotin Antibody Cocktail was added to the well and incubated for 1 hour at room temperature. After three wash cycles Streptavidin-PE was added to the wells and the plate was incubated 30 minutes at room temperature. The cytokine concentration was measured using a Bio-Rad analyzer (Bio-Rad, Germany).

### RNA isolation and real-time quantitative PCR

RNA isolation and cDNA construction were performed using a Cells-to-CT^TM^ Kit (Life Technologies, Carlsbad, CA, USA) according to the manufacturer's instructions. TaqMan PCR reagents for IL-6 (Hs00985639) and VEGFA (Hs00900055_m1) were purchased from ABI (Applied Biosystems, Foster, CA, USA). Quantitative Real-time PCR was carried out using TaqMan Master Mix reagents kit protocol with a StepOne Real-time PCR System (Life Technologies). The data were standardized against beta-actin gene expression using Pre-Developed TaqMan Assay Reagents (Applied Biosystems).

### Immunohistochemical staining of IL-6 in tumor specimens

Formalin-fixed, paraffin-embedded human tissues including renal cell carcinoma were processed for immunohistochemical staining with an anti-IL-6 antibody (ROCKLAND^TM^ Gilbertsville, PA). Sections (2 μm thick) were deparaffinized in three changes of xylene, hydrated through a descending series of ethanol. The endogenous peroxidase activity was blocked with methanol containing 0.5% hydrogen peroxide for 20 minutes. Then sections were treated at 4 °C overnight with primary antibodies for IL-6 (1:600 dilution). Subsequent reactions were carried out with the DAKO EnVision Detection System (Dako, Hamburg, Germany) according to the manufacturer's instructions.

### Immunohistochemical staining of IL-6R

For IL-6R staining on 786-O cells a Dako-REAL-EnVision HRP system was used (Dako, Hamburg, Germany). Cells were acetone fixed, peroxidase blocked and stained using an antihuman IL-6R antibody (1:25, R&D systems, Minneapolis, MN, USA) for one hour at room temperature. Second antibody (ready to use) and DAB staining was performed according to the manufacturer's instructions. Cells were HE counterstained. The primary antibody was omitted as negative control.

### MTT assay

For determination of cellular proliferation and viability, MTT (3-[4,5-dimethylthiazol-2-yl]-2,5-diphenyltetrazolium bromide) assays were performed using a Cell Proliferation Kit I (Roche Applied Science, Basel, Switzerland) according to the manufacturer's protocol. Briefly, 72 hours after incubation of the cells with TKIs and tocilizumab, the MTT labeling reagent was added to each well, which were then incubated for 4 hours at 37°C before the addition of the solubilization solution to each well. Spectrophotometrical absorbances of the samples were measured using a microplate reader. The absorbances were compared with those of non-treated cells.

### Measurement of VEGF using an enzyme-linked immunosorbent assay (ELISA)

786-O cells were treated by TKIs at various concentrations for two hours. The VEGF concentration in the supernatant of TKI treated 786-O cells with or without tocilizumab treatment were measured using a Human VEGF Quantikine ELISA kit (R&D systems, MN) according to the manufacturer's instructions.

### Western blotting analysis

786-O cells were treated with sorafenib with or without tocilizumab for 2 hours. Whole-cell protein was extracted from the cells treated with TKIs and/or tocilizumab and sodium dodecyl sulfate–polyacrylamide gel electrophoresis was carried out. Antibodies specific for phospho-AKT (p-AKT, Ser473), AKT, phospho-mTOR (p-mTOR, Ser2448), mTOR, phospho-STAT3 (p-STAT3, Tyr705), STAT3, phospho-4EBP1 (p-4EBP1,Thr37/46), phospho-p70S6K (p-p70S6K Ser411), phospho-S6 Ribosomal Protein (p-S6RP Ser235/236), phospho-NFκB (p-NFκB, Ser536), NFκB and HIF-2α were used as primary antibodies (Cell Signaling, Beverly, MA, USA). Anti-beta-actin antibody (SIGMA, St. Louis, MO, USA) was used as an internal control. Protein bands were visualized using ECL Advanced Western detection reagents (GE Healthcare, Buchinghamshire, UK), and imaged with a ChemiDoc XRS plus system (BIO-RAD, Hercules, CA, USA). Individual bands were quantified with Image Lab 2.0 software (BIO-RAD), and normalized against the control value (untreated cells).

### Tumor xenografts

All animal studies were conducted in compliance with Japanese animal use regulations and approval for these studies was obtained from the Committee on Animal Research of Fukushima Medical University. Six-week-old female BALB/C nu/nu nude mice (CLEA Japan, Inc.) were inoculated subcutaneously (s.c.) in the flank with 4 × 10^6^ 786-O cells (*n* = 20). The tumor-bearing mice were separated into four groups of five animals. One group received an i.p. injection of 100 mg antihuman IL-6 receptor (IL-6R) antibody, tocilizumab, three times a week, together with sorafenib in a daily of 10 mg/kg by oral administration as described previously [54]. The other groups received sorafenib alone, tocilizumab alone or phosphate-buffered saline (PBS; non-treated control group), respectively. Tumor size was measured and tumor volume was calculated by ellipsoid volume formulas (pi/6 x L x W x H) [55]. After treatment for 40 days, mice were sacrificed and the tumors were removed and used for histo-morphological analyses.

### Morphological and immunohistochemical examinations

Paraffin-embedded sections of tumors from the mouse xenograft models were prepared and stained with hematoxylin and eosin (HE). To detect activation of AKT-mTOR pathway, sections were stained with anti-CD31, anti-p-S6RP, anti-p-p70S5K and anti-p-4EBP1 antibodies (Cell Signaling Technology, Danvers, MA, USA). Staining was detected using a streptavidin-biotin kit (Nichirei, Trappes, France) according to the manufacturer's protocol.

### FDG-PET imaging

To evaluate a potential impact of tocilizumab on a TKI therapy of RCC, FDG-PET imaging in xenograft models of mice treated with sorafenib alone and the combination sorafenib with tocilizumab was performed. Xenograft tumors in nude mice were achieved as described above. To reinforce the effect observed in the xenograft experiments, the experimental group received 30 mg/kg of sorafenib 6 days a week (*n* = 8), for the duration of the study starting from time when the tumors reached 10 mm in diameter. For combination therapy (*n* = 7) tocilizumab (i.p. injection of 100 mg three times a week) was additionally administered. Mice were fasted for 6 hours prior to start of imaging session. FDG (obtained as an aliquot from daily clinical productions at Fukushima Medical University Hospital, 7–8 MBq per mouse, maximum volume of 200 μl) was administered to mice by a bolus injection via the tail vein. PET data were acquired in fully 3-dimensional (3-D) mode. Standard Uptake Values (SUV) were calculated for 3D regions of interest (ROI), using Inveon Research Workplace software (Siemens Medical Solutions). FDG-PET imaging was performed at baseline and at scheduled intervals on days 3 and 21 (each *n* = 3 or 4). Animals were sacrificed for radiologic-pathologic correlation according to the schedule.

### Statistical analysis

Determination of TKI treated cell proliferation, mRNA expression level and Western blot analysis were repeated in triplicate, and the results were expressed as the mean ± SD. The mRNA and protein expression levels as well as tumor volume from the mouse xenograft models were analyzed using a Wilcoxon rank sum test.

## Abbreviations

4EBP1: eukaryotic initiation factor 4E binding protein-1
AKT: AKT8 virus oncogene cellular homolog
ERK: extracellular Signal-regulated Kinase
FDG: fluorine-18 fluorodeoxyglucose
HIF: hypoxia-inducible factor
IL-6: interleukin 6
IL-6R: interleukin 6 receptor
JAK/STAT: janus kinase/signal transducer and activator of transcription
MAPK: mitogen-activated protein kinase
NFκB: nuclear factor κ B
mTOR: mammalian target of rapamycin
mTORC: mTOR complex
p706SK: p70S6 kinase
PDGFR: platelet-derived growth factor receptor
PET: positron emission tomography
RCC: renal cell carcinoma
S6RP: 40S ribosomal protein S6
SK1: sphingosine kinase-1
SUV: standardized uptake value
TKI: tyrosine kinase inhibitor
VEGF: vascular endothelial growth factor
VEGFR: vascular endothelial growth factor receptor.
